# Polysplenia syndrome with duodenal and pancreatic dysplasia in a Holstein calf: a case report

**DOI:** 10.1186/s12917-017-1213-2

**Published:** 2017-09-29

**Authors:** Daisuke Kondoh, Tomomi Kawano, Tomoaki Kikuchi, Kaoru Hatate, Kenichi Watanabe, Motoki Sasaki, Norio Yamagishi, Hisashi Inokuma, Nobuo Kitamura

**Affiliations:** 1 Division of Basic Veterinary Medicine, Obihiro University of Agriculture and Veterinary Medicine, Obihiro, Hokkaido Japan; 2 Division of Clinical Veterinary Medicine, Obihiro University of Agriculture and Veterinary Medicine, Obihiro, Hokkaido Japan

**Keywords:** Cattle, Duodenum, Dysplasia, Heterotaxy, Laterality disorder, Malposition, Pancreas, Polysplenia

## Abstract

**Background:**

Laterality disorders of the abdominal organs include situs inversus totalis that mirrors the arrangements of all internal organs and heterotaxy syndrome (situs ambiguus) in which the thoracic or abdominal organs are abnormally arranged. Heterotaxy is often accompanied by multiple congenital malformations, and it generally comprises asplenia and polysplenia syndromes. To our knowledge, polysplenia syndrome has been reported in only three cattle, and computerized tomographic (CT) images of these animals were not obtained.

**Case presentation:**

A six-month-old Holstein heifer had ruminal tympani and right abdominal distension. CT imaging showed that the rumen occupied the right side of the abdominal cavity, the omasum and abomasum occupied the left ventral side and the liver was positioned on the left. The colon and cecum were located at the left dorsum of the cavity, and the left kidney was located more cranially than the right. Postmortem findings revealed two spleens attached to the rumen. Significantly, the duodenum was too short to be divided into segments, except the cranial and descending parts, or flexures, except the cranial flexure, and the pancreas, which lacked a left lobe, was covered with mesojejunum. The liver comprised a relatively large right lobe and a small left lobe without quadrate and caudate lobes. The caudal vena cava that connected to the left azygous vein passed irregularly through the aortic hiatus of the diaphragm, and the common hepatic vein without the caudal vena cava passed through the caval foramen. Although the lungs and heart were morphologically normal, the right atrium received three major systemic veins. Polysplenia syndrome was diagnosed based on the CT and postmortem findings.

**Conclusion:**

We defined the positions of the abdominal organs and morphological abnormalities in various organs of a calf with polysplenia syndrome based on CT and postmortem findings. These findings will improve understanding of the malpositioning and malformations that can occur in the organs of cattle with polysplenia syndrome.

**Electronic supplementary material:**

The online version of this article (10.1186/s12917-017-1213-2) contains supplementary material, which is available to authorized users.

## Background

Laterality disorders are atypical arrangements of internal organs, including situs inversus totalis and heterotaxy (situs ambiguus). Situs inversus totalis is a condition in which all thoracic and abdominal organs mirror the normal arrangement, and in humans it is closely associated with primary ciliary dyskinesia, also known as immotile cilia syndrome. Heterotaxy is an abnormal arrangement of the thoracic or abdominal organs that is often accompanied by multiple congenital malformations, especially cardiovascular malformations that are associated with high morbidity rates in humans [1, 2]. Heterotaxy generally comprises asplenia (right isomerism) and polysplenia (left isomerism) syndromes but exceptions are numerous, and the spectrum of abnormalities seems to overlap [2]. The estimated prevalence of situs inversus totalis and of heterotaxy with cardiovascular malformation in humans is 1 per 8000–25,000 individuals and 1 per 10,000 live births, respectively [1]. Situs inversus totalis has been found in several dogs [3], cats [4], horses [5] and pigs [6], and heterotaxy in two dogs [7, 8] and a sheep [9] has also been reported.

A few reports have described laterality disorders of the abdominal organs in cattle [10–13]. As far as we can ascertain, only one of six reported cattle with laterality disorders of the abdominal organs had situs inversus totalis [12]. The other five had heterotaxy, consisting of polysplenia and asplenia syndromes in three and two, respectively [10, 11, 13], accompanied by cardiovascular malformations (irregular continuation of the caudal vena cava). These findings indicated that the features of heterotaxy in cattle are at least partly similar to those in humans. However, the location of abdominal organs in live cattle with laterality disorders has not been described in detail.

Here, we show the first computerized tomographic (CT) images of a calf with polysplenia syndrome and detailed postmortem findings of complicating duodenal and pancreatic dysplasia.

## Case presentation

### Animal and clinical findings

**Fig. 1:**
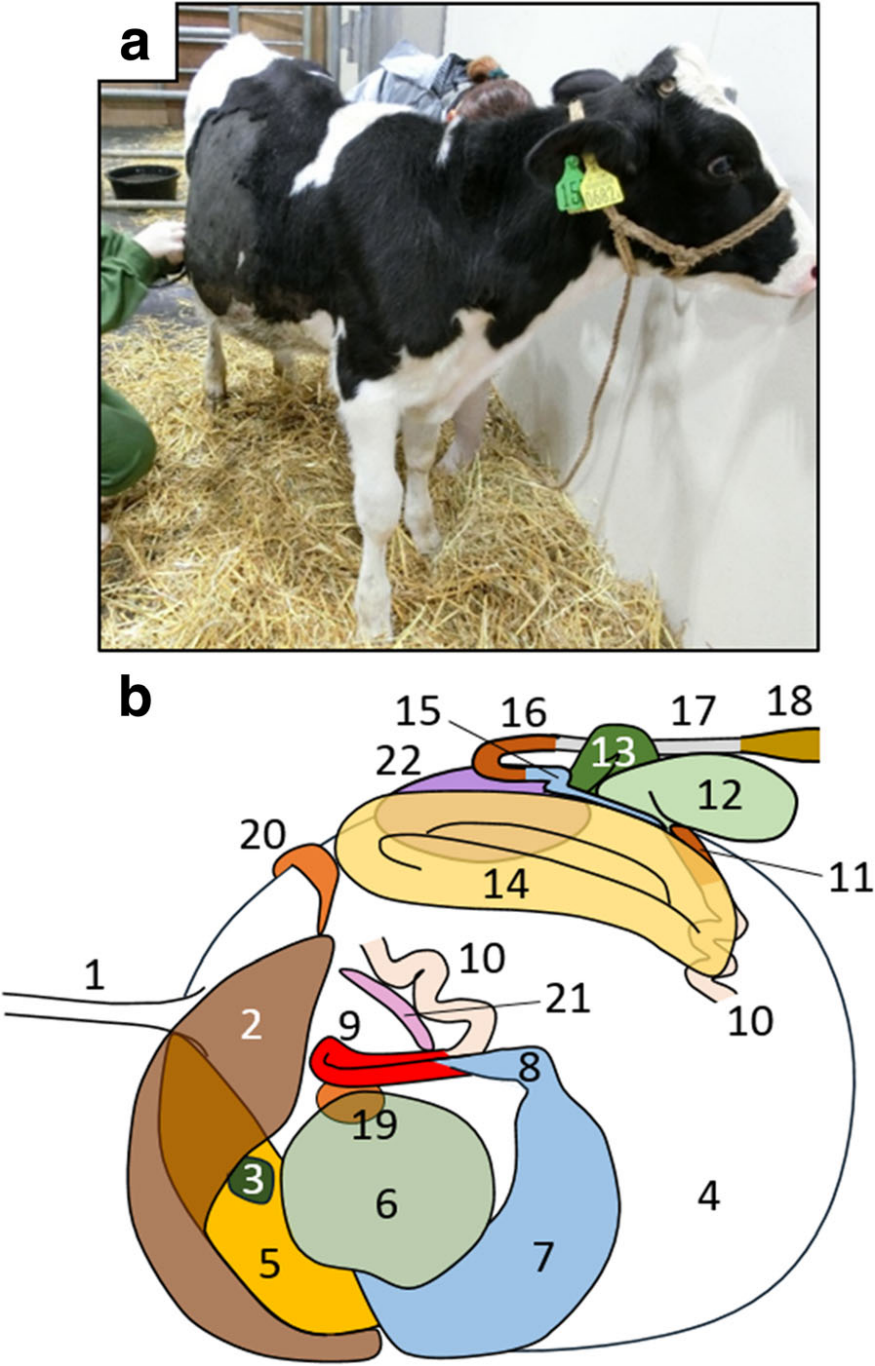
Six-month-old Holstein calf with polysplenia syndrome. **a** Initial findings of calf with right abdominal distension. **b** Schema of left lateral view of abdominal organ locations. Left of panel, cranial to body; upper, dorsal. 1, esophagus; 2, liver; 3, gall bladder; 4, rumen; 5, reticulum; 6, omasum; 7, abomasum; 8, pylorus; 9, duodenum; 10, jejunum; 11, ileum; 12, cecum; 13, proximal loop of colon; 14, spiral loop of colon; 15, distal loop of colon; 16, transverse colon; 17, descending colon; 18, rectum; 19, ventral spleen; 20, dorsal spleen; 21, pancreas; 22, left kidney

A six-month-old Holstein heifer with ruminal tympani was initially examined by a local veterinarian (day 1) who found right abdominal distension (Fig. 1a), and ruminal sounds from the right flank upon auscultation. Rectal palpation revealed the rumen and kidney on the right side. Ultrasound showed that the liver was located on the left side between the 5th and 8th intercostal spaces. Situs inversus was suspected, and the calf was transferred to the Animal Teaching Hospital, Obihiro University of Agriculture and Veterinary Medicine for further examination on day 9. At that time, the general health status of the calf appeared normal. Rectal temperature, heart rate and respiratory rate were 39.2 °C, 96 bpm and 48 breaths/min, respectively. Abdominal auscultation identified three ruminal movements every 2 min and a “ping” sound on the right flank. The findings of rectal palpation were similar to those on day 1.

Hematological findings of the red blood cell count (738 × 10^4^/μL), hemoglobin concentration (9.1 g/dL), packed cell volume (27.7%) and white blood cell count (10,800/μL) were normal. Blood chemistry showed normal values for aspartate aminotransferase activity (91 U/L), blood urea nitrogen (6.8 mg/dL), sodium (142 mEq/L), potassium (4.5 mEq/L), chloride (101 mEq/L) and total protein (6.6 g/dL). The general health status of the calf did not change until euthanasia on day 35.

Figure 1b and Additional file 1: Figure S1 summarize a conceivable arrangement of the major abdominal organs in this calf based on the CT and postmortem findings described below.

### CT findings

**Fig. 2:**
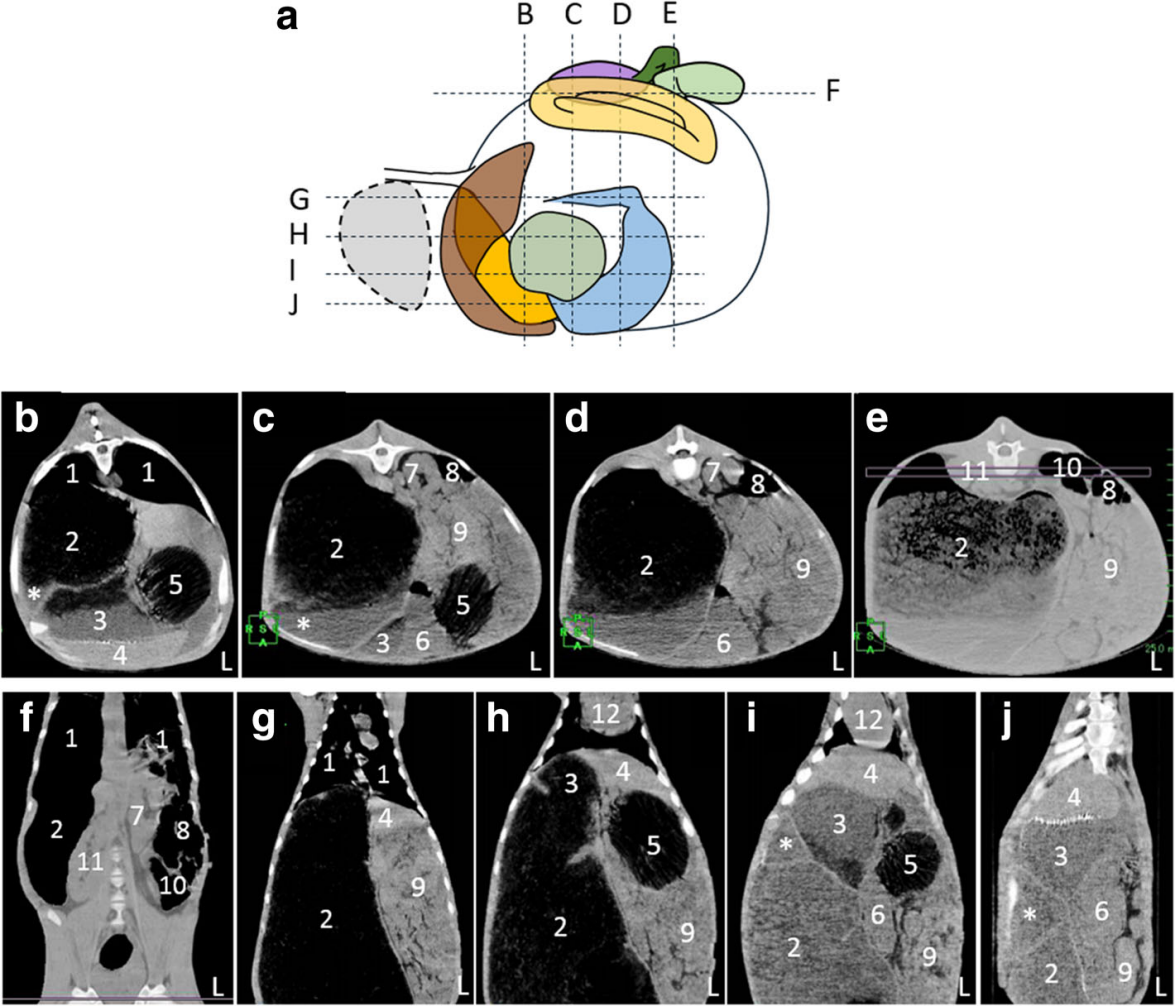
Computerized tomography (CT) images of Holstein calf with polysplenia syndrome. **a** Schema of tomographic parts in abdominal cavity. Dashed lines (B-J) correspond to panels (B-J). (Also see Fig. 1b to compare organs in this schema). Dashed circle, heart. **b**-**e** Coronal CT images. Left of panels, right side of body; upper, dorsal side. **f**-**j** Horizontal CT images. Left of panels, right side of body; upper, cranial side. L, left side of body; 1, lung; 2, rumen; 3, reticulum; 4, liver; 5, omasum; 6, abomasum; 7, left kidney; 8, spiral loop of colon; 9, jejunum; 10, cecum; 11, right kidney; 12, heart. *Saccus cranialis

CT images showed that the rumen occupied the right side of the abdominal cavity (Fig. 2, Additional file 1: Figure S2). The saccus cranialis was undeveloped (Fig. 2b–j), and other compartments including saccus dorsalis and saccus ventralis were not detected in the rumen (Fig. 2d, e). The reticulum was located anterior to the rumen and contacted the diaphragm, and the omasum and abomasum occupied the left ventral side of the abdominal cavity (Fig. 2b–d, h–j). The colon and cecum were positioned at the most dorsal part of the abdominal cavity and to the left side of the left kidney (Fig. 2c–f). The liver was located at the most cranial position in the left side of the abdominal cavity and was surrounded by the diaphragm, reticulum and omasum (Fig. 2b, g–j). The left kidney was located more cranial than the right (Fig. 2c–f).

### Postmortem findings

The calf was euthanized under anesthesia via an intravenous injection of xylazine followed by thiamylal sodium. The abdominal and thoracic organs were sequentially examined in detail throughout necropsy and isolated to evaluate morphological abnormalities. Various organs were fixed in Bouin’s fluid, and paraffin-embedded sections were processed for histopathological analysis.

#### Organs in the abdominal cavity

**Fig. 3:**
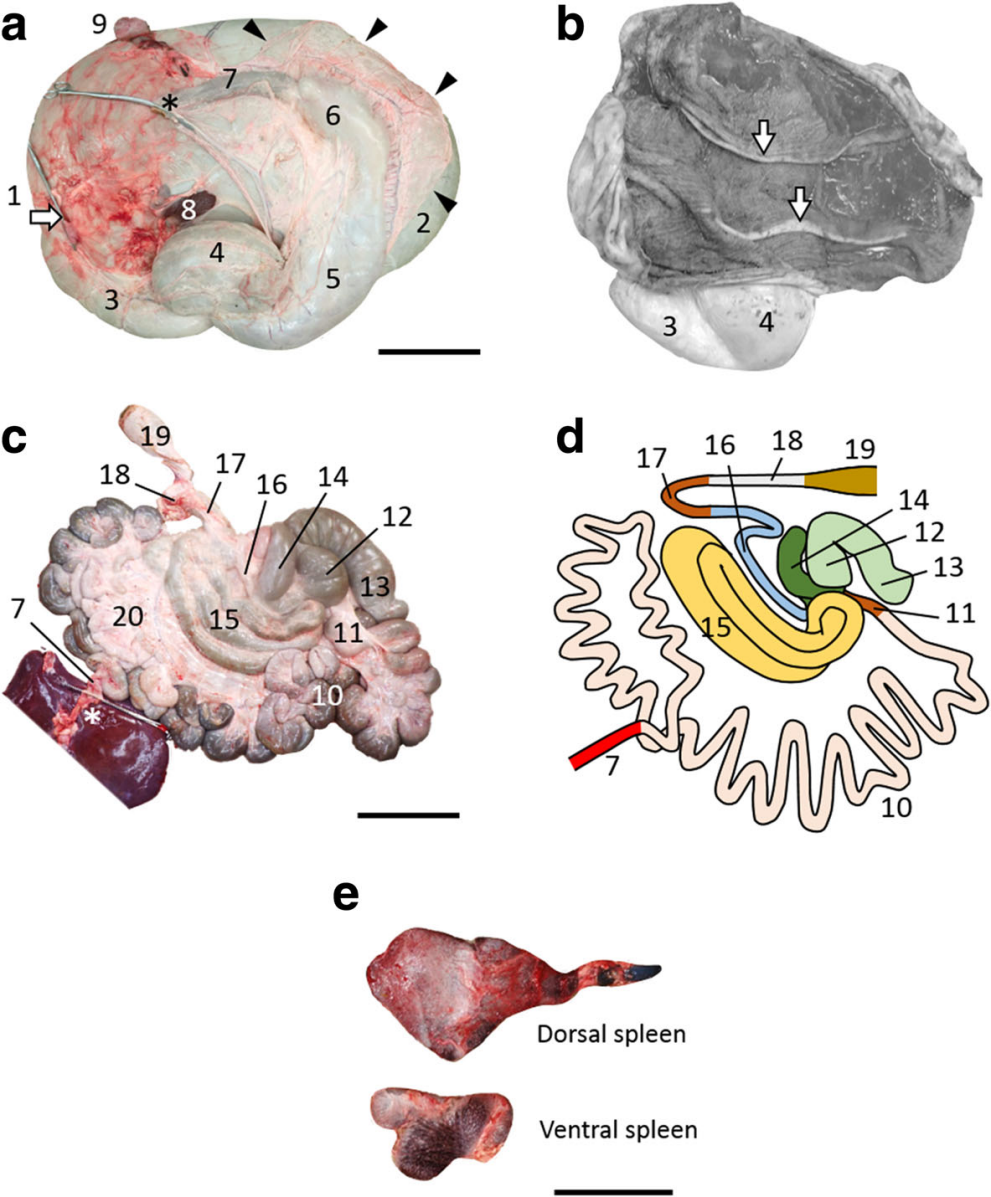
Morphology of digestive tract and attached spleens. **a** Left lateral view of the isolated digestive tract from esophagus to duodenum and spleens. Arrowheads indicate greater omentum. *Severed part of duodenum conforms to asterisk in panel (**c**). Arrow, severed part of esophagus. Left of panels (**a**-**d**), cranial; upper, dorsal. **b** Internal structure of rumen with two pillars (arrows). **c** Left lateral view of isolated digestive tract from duodenum to transverse colon and liver. *Severed part of duodenum conforms to asterisk in panel (**a**). **d** Schema of lower digestive tract according to panel (**c**). **e** Morphology of dorsal and ventral spleens. Bar = 20 (**a** and **c**) and 10 (**e**) cm. 1, esophagus; 2, rumen; 3, reticulum; 4, omasum; 5, abomasum, 6, pylorus; 7, duodenum; 8, ventral spleen; 9, dorsal spleen; 10, jejunum; 11, ileum; 12, body of cecum; 13, apex of cecum; 14, proximal loop of colon; 15, spiral loop of colon; 16, distal loop of colon; 17, transverse colon; 18, descending colon; 19, rectum; 20, mesojejunum

The esophagus joined the rumen at the relative left cranial position and opened immediately dorsal to the reticulum (Fig. 3a). Sulci of the rumen were not significant, and only two longitudinal pillars were detected at the mucosal side (Fig. 3b). The reticulum that continued anteroventrally from the rumen was recognized the left lateral view of the stomach, and the omasum and abomasum were located at the left ventral side of the rumen (Fig. 3a). The duodenum that continued from the pylorus and ran cranially (Fig. 3a), turned caudally near the porta hepatis (Fig. 3a, c; asterisks) to form the cranial flexure, but not the sigmoid loop, and then joined the jejunum without flexures (Fig. 3c, d, Additional file 1: Figure S3). Therefore, the cranial and descending parts of the duodenum were identified, but the caudal and ascending parts were not. The jejunum was distinguished from the duodenum by the presence of the mesojejunum. The cecum and colon occupied the left dorsal region of the abdominal cavity, with the apex of the cecum being located more medially than the spiral loop of the colon (Figs. 2e, 3c, d, Additional file 1: Figure S3). Proximal, spiral and distal loops were found in the ascending colon, and a loose distal loop continued to the transverse colon (Fig. 3c, d, Additional file 1: Figure S3). Dorsal and ventral spleens were attached to the cranial part of the rumen (Fig. 3a, e). The ventral spleen was located on the left between the rumen and the omasum, and the dorsal spleen was located at the midline of the rumen (Fig. 3a).

The size of lesser omentum was normal, but the greater omentum was very small (Fig. 3a). The lessor omentum was attached to the omasum and liver as usual, although it was located at left side of the body. The greater omentum was attached to the greater curvature of abomasum, spread caudally, immediately turned cranially and ended at the left side of rumen.

**Fig. 4:**
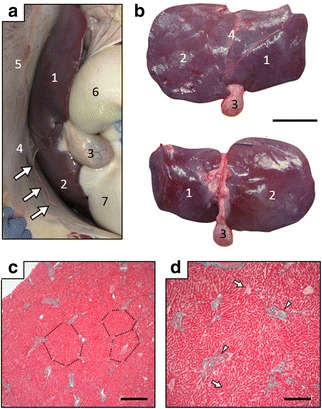
Morphology and location of liver. **a**. Left lateral view of liver located at cranioventral region of abdominal cavity. Arrows, round ligament. Left of panels, cranial to body; upper, dorsal. **b** Diaphragmatic (upper) and visceral (lower) surfaces of liver. **c** and **d** Histological properties of liver. Dashed lines indicate hepatic lobules in panel (**c**). Arrows and arrowheads, central veins and portal canals, respectively, in panel (**d**). Masson-Goldner staining. Bars = 10 cm (**b**) and 500 (**c**) and 200 (**d**) μm. 1, left lobe; 2, right lobe, 3, gall bladder; 4, falciform ligament; 5, diaphragm; 6, rumen; 7, reticulum

The liver was located at the left anteroventral area of the abdominal cavity, and the falciform and round ligaments as well as the gall bladder were found in the left lateral view of the cavity (Fig. 4a). The relatively large right lobe and small left lobe were identified by the origin of the falciform ligament, but the caudate lobe was not distinguished (Fig. 4). Because the fissure for round ligament (fissure ligamentum teretis) entered the nearby region of the fossa for gall bladder (fossa vesicae felleae), the quadrate lobe was not recognized (Fig. 4b).

**Fig. 5:**
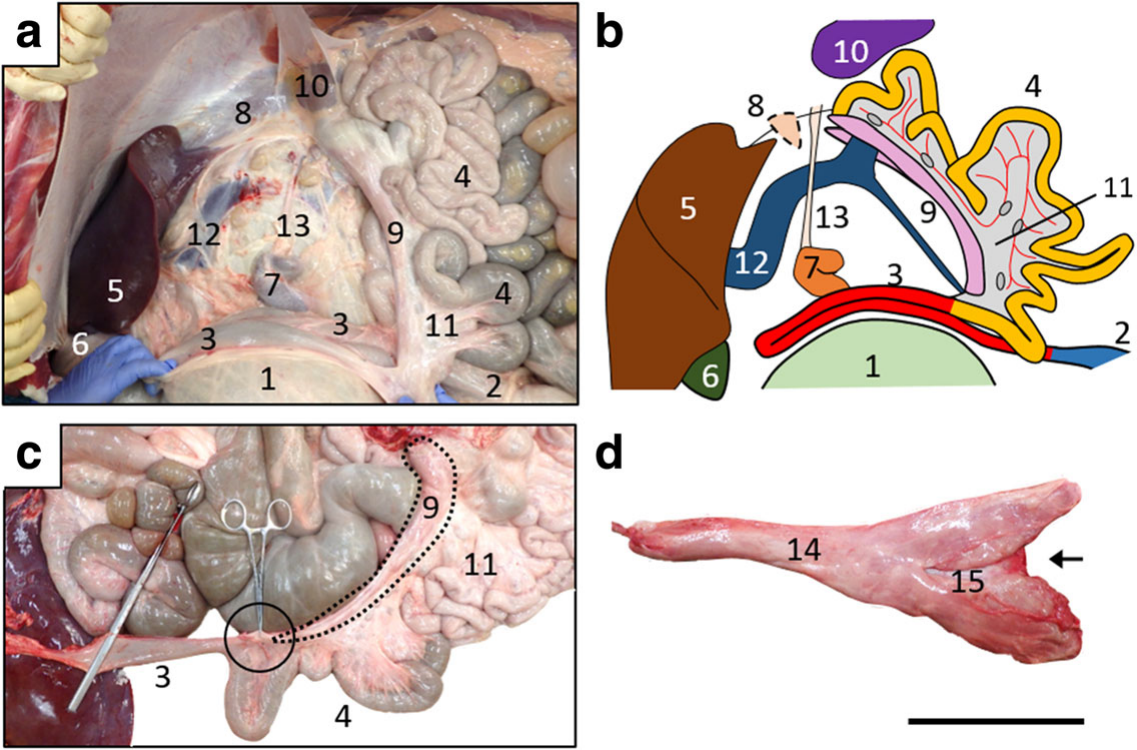
Location of organs surrounding pancreas and morphology of pancreas. **a** Left lateral view of organs and vessels located at craniodorsal region of abdominal cavity. Left of panels (**a**-**c**), cranial; upper, dorsal. **b** Schema of positional relationships among pancreas, duodenum, jejunum, liver and portal vein according to panel (**a**). **c** Left lateral view of isolated duodenum, jejunum, pancreas and mesojejunum. Dashed line, pancreas; solid circle, opening of accessory pancreatic duct. **d** Morphology of pancreas. Arrow, pancreatic notch. Bar = 10 cm. 1, omasum; 2, pylorus; 3, duodenum; 4, jejunum; 5, liver; 6, gall bladder; 7, ventral spleen; 8, dorsal spleen; 9, pancreas; 10, left kidney; 11, mesojejunum; 12, portal vein; 13, splenic artery; 14, right lobe of pancreas; 15, body of pancreas

An elongated, pole-like pancreas was abnormally covered with mesojejunum (Fig. 5a–c, Additional file 1: Figure S4) and comprised the body and the right, but not the left lobe (Fig. 5d). The hepatic portal veins passed through the pancreatic notch (Fig. 5a, b, Additional file 1: Figure S4), and the accessory pancreatic duct that joined the descending part of the duodenum protruded from the right lobe (Fig. 5c).

**Fig. 6:**
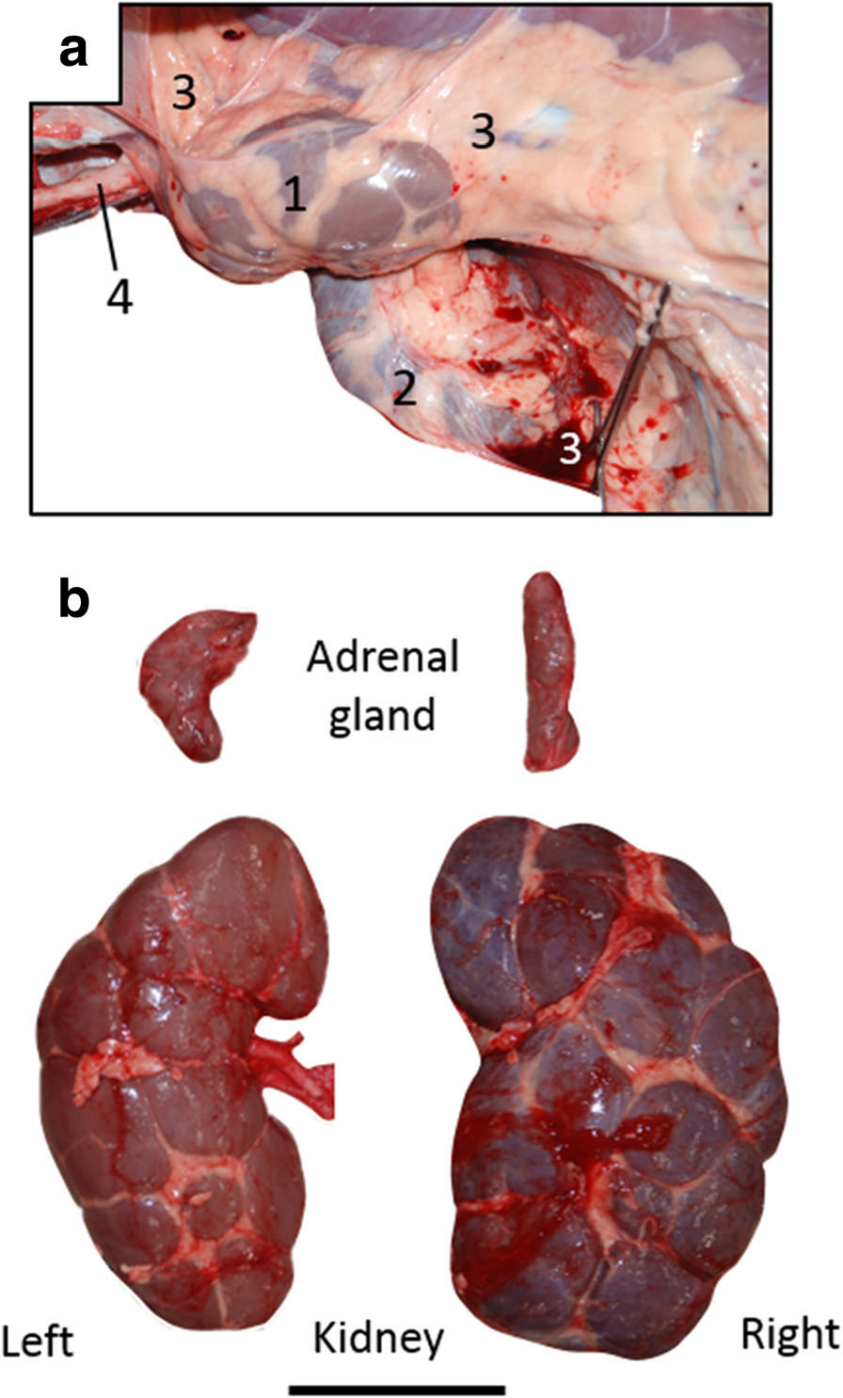
Morphology and location of right and left kidneys. **a** Left lateral view of retroperitoneal organs. Left of panel, cranial; upper, dorsal. **b** Morphology of right and left kidneys and adrenal glands. Bar = 10 cm. 1, left kidney; 2, right kidney; 3, peritoneum; 4, abdominal aorta

Both the left and right kidneys were retroperitoneal, and the smaller left kidney was secured more cranially than the right (Fig. 6a and b). The shape of the right adrenal gland was irregularly pole-like, whereas the left was comma-shaped as normal (Fig. 6b). The positions and morphological findings of other urogenital organs were normal.

The gastrointestinal tract, liver (Fig. 4c, d), spleen, pancreas, kidney, adrenal gland and uterus were histologically normal.

#### Diaphragm, vascular system and organs in the thoracic cavity

**Fig. 7:**
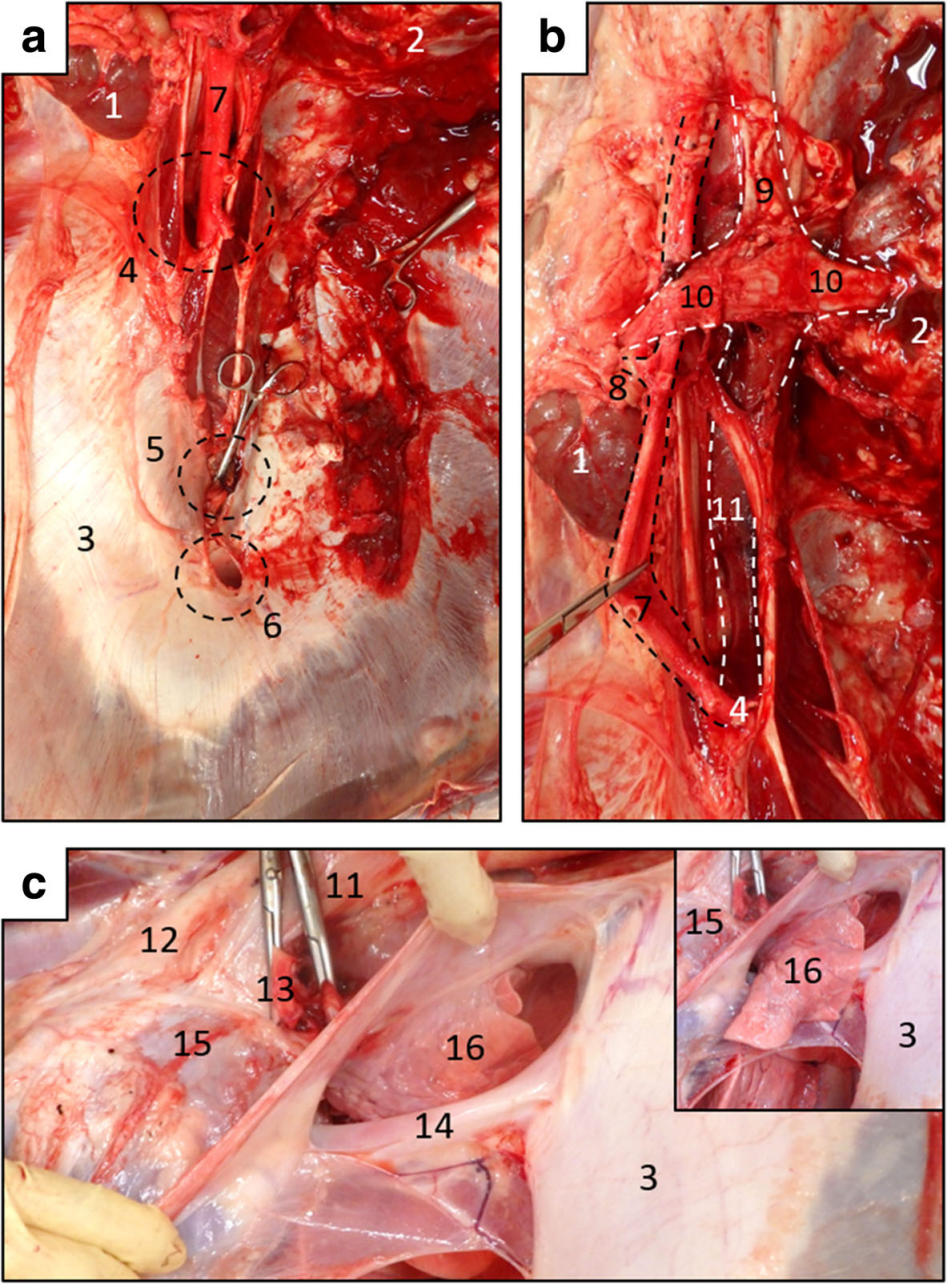
Morphology of diaphragm and major vascular system. **a** Caudal view of diaphragm. Left of panel, left of body; upper, dorsal. **b** Ventral view of abdominal aorta and caudal vena cava that joins left azygous vein. Black and white dashed lines, arteries and veins, respectively. Left of panel, left of body; upper, caudal. **c** Left lateral view of common hepatic vein that passes through caval foramen into thoracic cavity. Accessory lobe of right lung is evident dorsally. Insert, accessory lobe covering common hepatic vein. Left of panel, cranial to body; and upper, dorsal. 1, left kidney; 2, right kidney; 3, central tendon; 4, aortic hiatus; 5, esophageal hiatus; 6, caval foramen; 7, abdominal aorta; 8, renal artery; 9, caudal vena cava; 10, renal vein; 11, left azygos vein; 12, thoracic aorta; 13, pulmonary trunk; 14, common hepatic vein; 15, left atrium; 16, accessory lobe of right lung

The diaphragm possessed an aortic hiatus between the left and right crura, an esophageal hiatus through the ventral region of the right crus, and a caval foramen through the central tendon (Fig. 7a). The celiac, cranial mesenteric, renal and caudal mesenteric arteries were normally derived from the abdominal aorta. The caudal vena cava that connected with the renal veins irregularly joined the left azygos vein and passed through the aortic hiatus of the diaphragm (Fig. 7b). Several hepatic veins joined to form the common hepatic vein without a caudal vena cava, and it passed through the caval foramen (Fig. 7c).

**Fig. 8:**
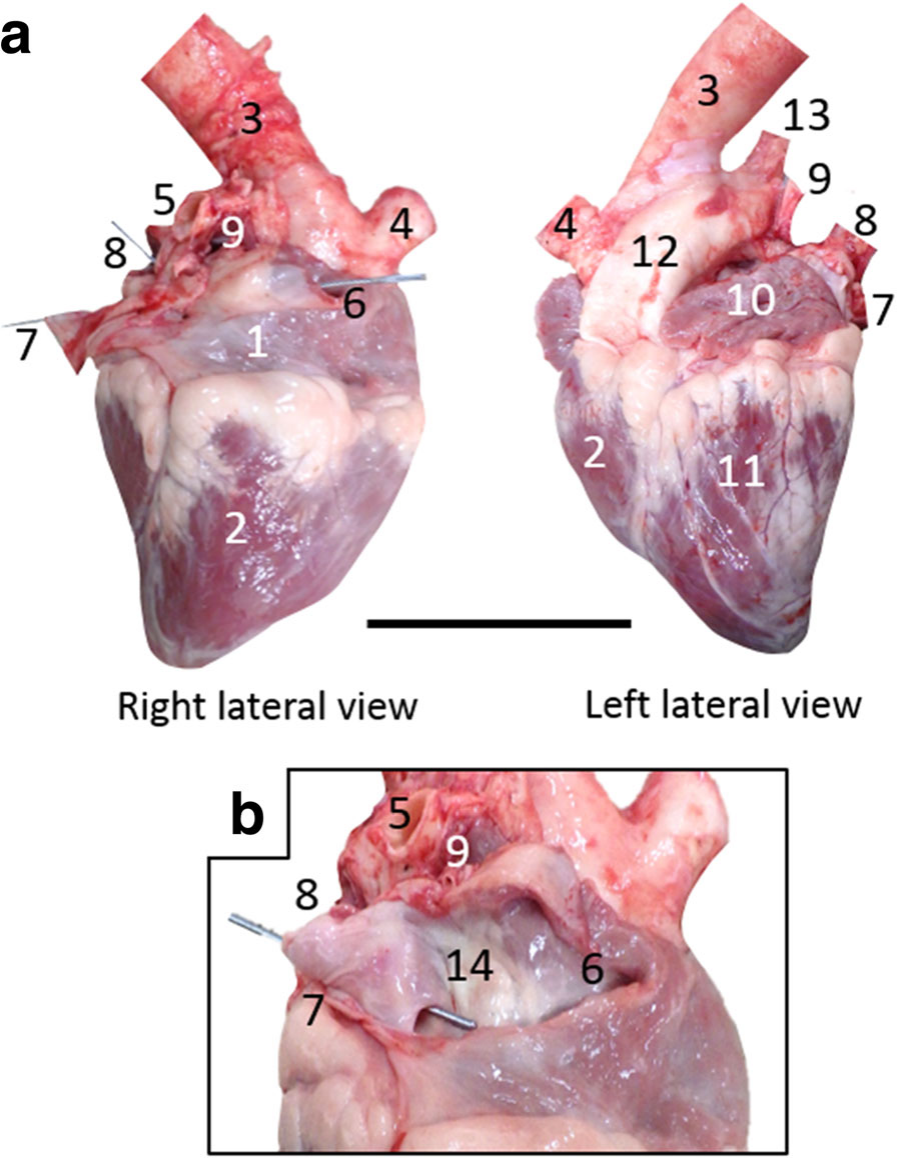
Cardiac morphology. **a** Right and left lateral views of isolated heart. Bar = 10 cm. **b** Internal structures of right atrium. 1, right atrium; 2, right ventricle; 3, thoracic aorta; 4, brachiocephalic trunk; 5, right pulmonary artery; 6, cranial vena cava; 7, common hepatic vein; 8, left azygos vein; 9, pulmonary veins; 10, left atrium; 11, left ventricle; 12, pulmonary trunk; 13, left pulmonary artery; 14, sinus venarum cavarum

**Fig. 9:**
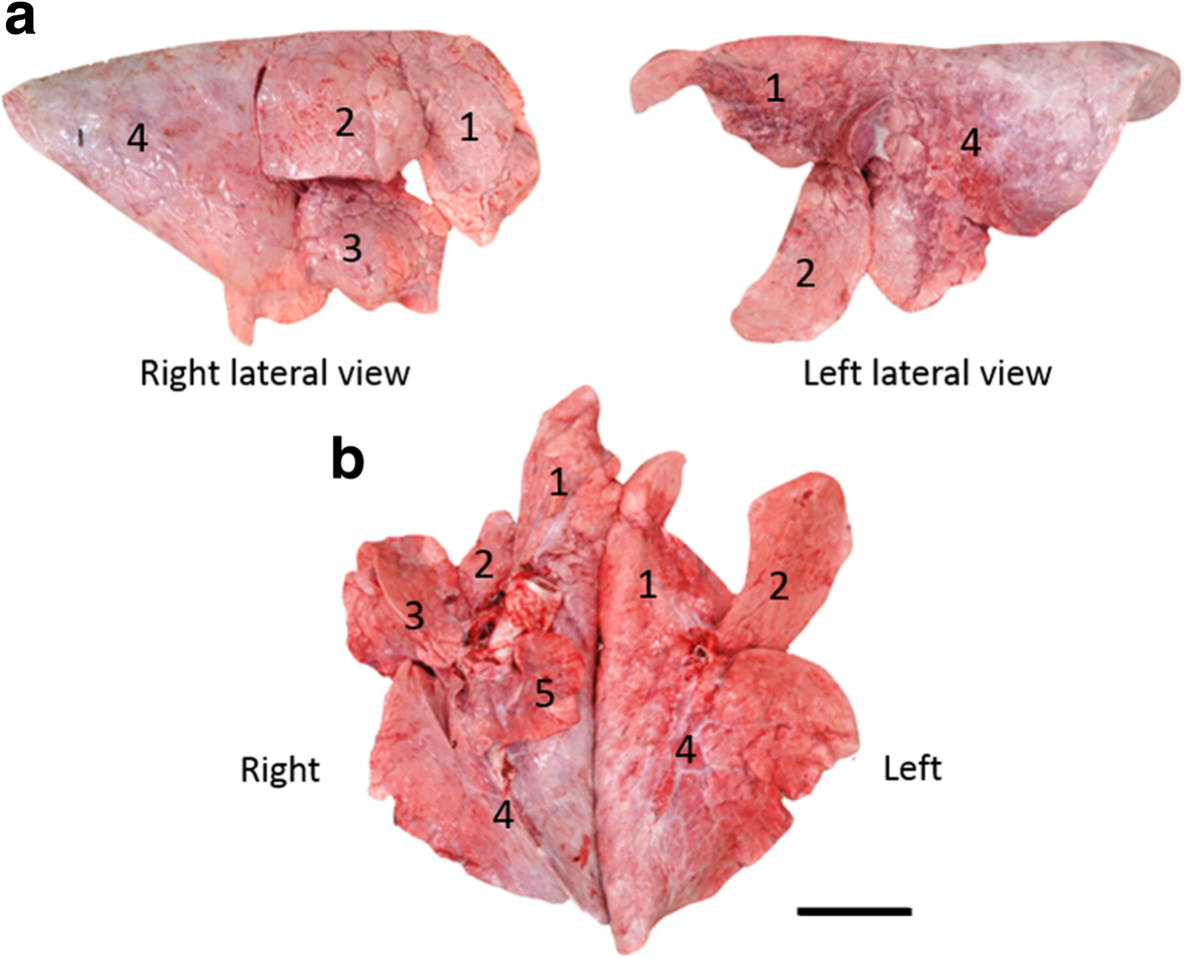
Morphology of right and left lungs. **a** Lateral views of isolated right and left lungs. **b** Ventral view of isolated lungs. Bar = 10 cm. 1, cranial part of cranial lobe; 2, caudal part of cranial lobe; 3, middle lobe; 4, caudal lobe; 5, accessory lobe

The position and morphological findings of the atria and ventricles were normal except for the venous input pathway to the right atrium (Fig. 8a), which received the major systemic cranial vena cava, left azygos and common hepatic veins. The anastomosis of the cranial vena cava and the common hepatic vein formed the sinus venarum cavarum (Fig. 8b). On the other hand, the large azygos vein opened into a distended coronary venous sinus (Fig. 8b). The position and morphological findings of the lungs were normal (Fig. 9).

**Fig. 10:**
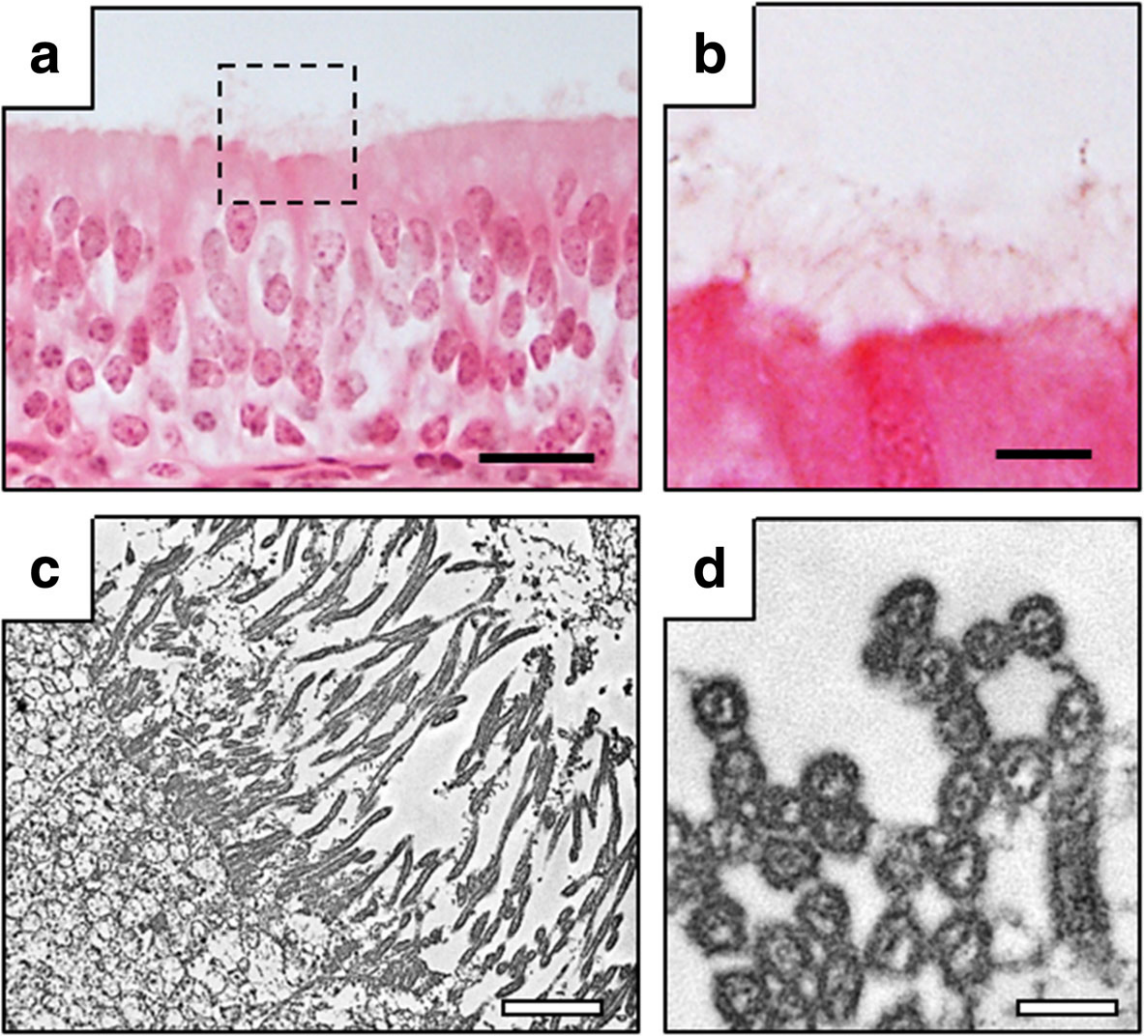
Histology of respiratory epithelium in trachea of Holstein calf with polysplenia syndrome. **a** Whole image of respiratory epithelium. Dashed box, region shown in panel (**b)** at high magnification. Hematoxylin-eosin staining. **b** cilia of respiratory epithelium. **c** and **d** Back transmission electron microscopic features of cilia, after paraffin embedding. Bars = 20 (**a**), 5 (**b**), 2 (**c**) and 0.3 (**d**) μm

The heart, lung and trachea were histologically normal. Light and electron microscopy showed that the respiratory epithelium had the cilia (Fig. 10).

## Discussion

Atypical arrangements of internal organs are generally categorized as situs inversus totalis and heterotaxy (situs ambiguus), and the latter essentially comprises asplenia and polysplenia syndromes. The present study found situs inversus of the abdominal organs, situs solitus of the thoracic organs and two spleens in a six-month-old Holstein calf, indicating a diagnosis of polysplenia syndrome. Although polysplenia and asplenia syndromes are generally known as left and right isomerism, respectively, in humans, both are often atypical and are accompanied by multiple congenital malformations [1, 2]. The morphological features of the liver, lungs and atria, as well as the position of the apex of the cecum without isomerism in this calf differed from those typical of human polysplenia syndrome. Further studies are needed to compare the characteristics of polysplenia syndrome between cattle and humans.

Three reports have described cattle with two spleens [10, 11, 13], and among six cattle described to date, three had atypically arranged abdominal organs (stomach and liver) indicating heterotaxy, namely, polysplenia syndrome. However, none of these animals had isomerism of the lungs and atria. On the other hand, among three cattle without spleen [11], two had atypically arranged abdominal organs [11] that was recognized as heterotaxy, namely asplenia syndrome. Both of these, as well as a remaining one, had right isomerism of the lungs. These facts suggest that the features of polysplenia syndrome in cattle differ from those in humans with left organ isomerism [8, 9], whereas asplenia syndrome in cattle might be similar to that in humans with typically right isomerism [1, 2]. In addition, cardiovascular malformations accompany not only polysplenia or asplenia syndromes in cattle with situs inversus, but also those with situs solitus of the abdominal organs regardless of having no or two spleens [11]. These facts indicate that association rules are not strict among the number of spleens, atypically arranged abdominal organs and cardiovascular malformations, and that the mechanism through which heterotaxy is generated seems complex and varied, at least in cattle.

Although severe dysplasia of duodenum and pancreas has not been reported as a complicating condition of polysplenia syndrome in cattle, a malformed annular or short pancreas and intestinal malrotation have been identified in several humans with polysplenia syndrome [14–17]. The present findings indicate that polysplenia syndrome in cattle is also accompanied by multiple organ malformations including duodenal and pancreatic dysplasia.

The calf described herein had abnormalities of the stomach, intestine, liver, kidneys, spleen and cardiovascular system, which, except for severe dysplasia of duodenum and pancreas, seemed broadly similar to those of the three previously described cattle with polysplenia syndrome [10, 11, 13], but there are also some relatively-small differences among these four cattle. Caudal vena cava continued to right and left azygos veins in two cattle [11, 13] and the calf reported herein, respectively, while that in the remaining one [10] joined common hepatic vein in the thoracic cavity. Two cattle with polysplenia syndrome [10, 11] possessed four lobes of liver with mirror image, while only three and two lobes with normal image were detected in the cow reported by Boos et al. [13] and the calf in the present case, respectively. In addition, Boos et al. [13] described some histological abnormalities of liver in the cow with polysplenia syndrome. Both two spleens were located at left side of rumen in two cattle [10, 11], while each spleen was attached to the left and right sides in a remaining cow [13], unlike the calf described herein. These differences indicate that various clinical conditions are recognized in cattle with polysplenia syndrome.

## Conclusion

The present CT and postmortem findings of a calf with laterality disorders allowed a detailed study of the abdominal organ positions (Fig. 1b) as well as of morphological abnormalities in various abdominal organs and the cardiovascular system. Our findings improve understanding of the malpositions and various types of malformations among the abdominal and thoracic organs of cattle with polysplenia syndrome.

## Additional file

Additional file 1: Figure S1. Schematic comparison of abdominal organs between calf presented herein (**A**) and general cattle (**B** and **C**), in association with Fig. 1. 1–22, corresponding to Fig. 1; 23, right kidney; 24, spleen; 25, Grooves of rumen. **Figure S2.** Comparison of computerized tomography (CT) images between calf presented herein (**A** and **C**) and general cattle (**B and D**), in association with Fig. 2. 1–6 and asterisks; corresponding to Fig. 2. **Figure S3.** Schematic comparison of digestive tracts between calf presented herein (**A**) and general cattle (**B**), in association with Fig. 3. Numbers are corresponding to Fig. 3. **Figure S4.** Schematic comparison of organs surrounding pancreas between calf presented herein (**A**) and general cattle (**B**), in association with Fig. 5. 1–13, corresponding to Fig. 3 (except 11, coalesced mesojejunum and mesocolon); 14–16, Body, right robe and left lobe of pancreas, respectively. (PDF 1156 kb)

## Abbreviation

CT: Computerized tomographic
