# Supplementary material for: Polysplenia syndrome with duodenal and pancreatic dysplasia in a Holstein calf: a case report
Source: BMC Vet Res. 2017 Sep 29;13:292. doi: 10.1186/s12917-017-1213-2 (PMC5622422; doi:10.1186/s12917-017-1213-2)
Supplement: Additional file 1: Figure S1. — Schematic comparison of abdominal organs between calf presented herein (A) and general cattle (B and C), in association with Fig. 1. 1–22, corresponding to Fig. 1; 23, right kidney; 24, spleen; 25, Grooves of rumen. Figure S2. Comparison of computerized tomography (CT) images between calf presented herein (A and C) and general cattle (B and D), in association with Fig. 2. 1–6 and asterisks; corresponding to Fig. 2. Figure S3. Schematic comparison of digestive tracts between calf presented herein (A) and general cattle (B), in association with Fig. 3. Numbers are corresponding to Fig. 3. Figure S4. Schematic comparison of organs surrounding pancreas between calf presented herein (A) and general cattle (B), in association with Fig. 5. 1–13, corresponding to Fig. 3 (except 11, coalesced mesojejunum and mesocolon); 14–16, Body, right robe and left lobe of pancreas, respectively. (PDF 1156 kb) [file 12917_2017_1213_MOESM1_ESM.pdf]

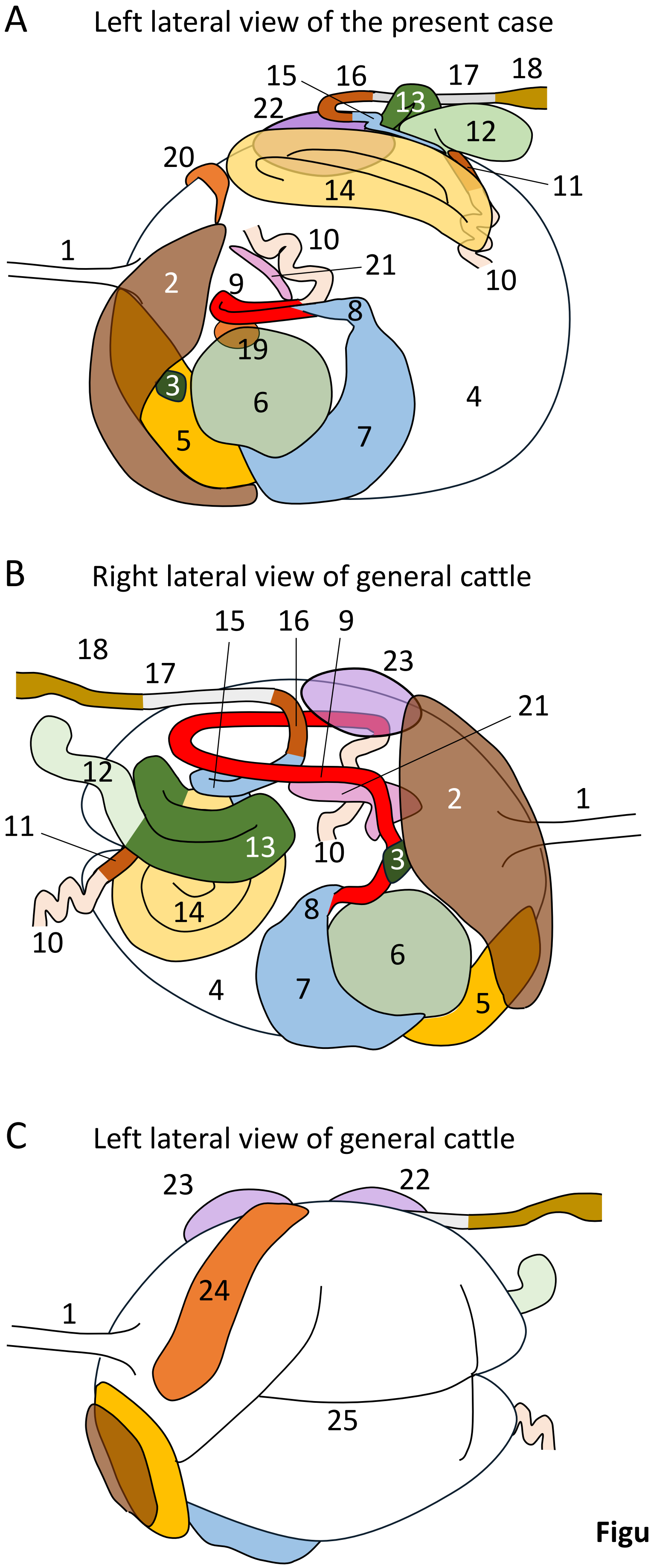
A
Left lateral view of the present case
15
16
17
18
22
13
12
20
11
14
10
1
21
2
9
10
8
19
3
6
4
5
7
B
Right lateral view of general cattle
15
16
9
18
23
17
21
12
2
1
11
13
10
3
14
8
10
6
4
7
5
C
Left lateral view of general cattle
23
22
24
1
25

Figure S1

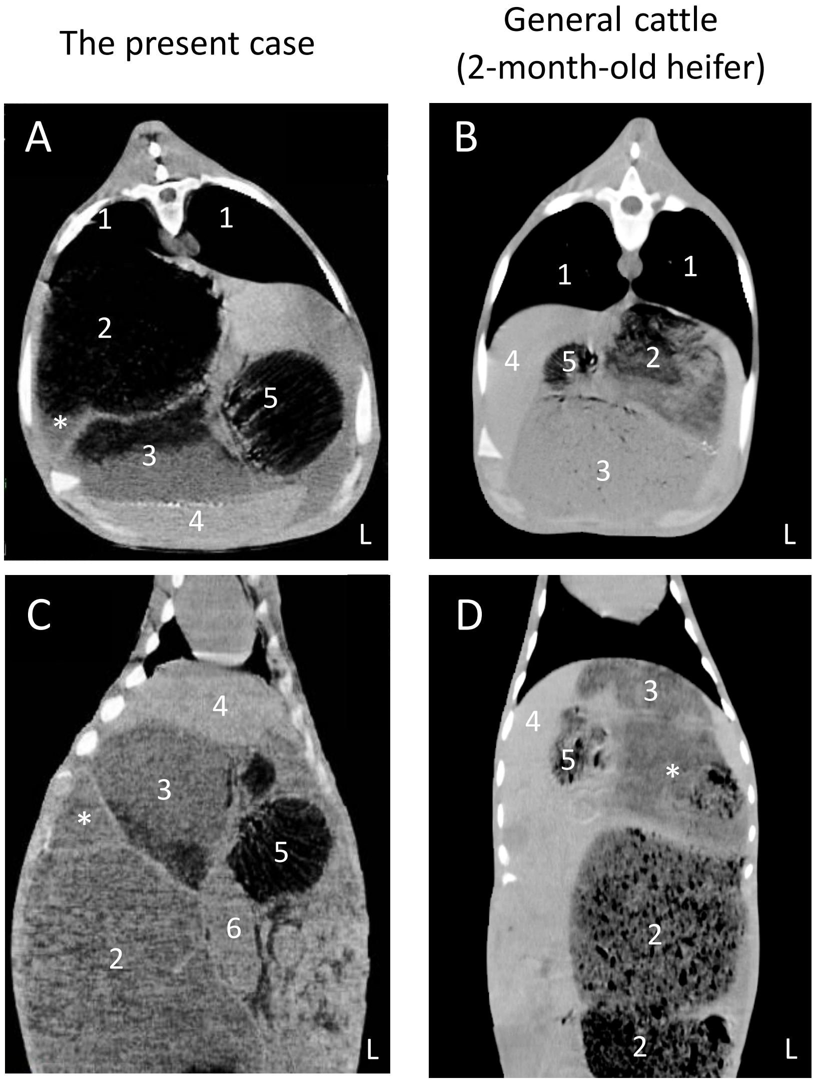

The present case
General cattle
(2-month-old heifer)
A
1
1
2
*
5
3
4
L
B
1
1
4
5
2
3
L
C
4
3
*
5
6
2
L
D
3
4
5
*
2
2
L

Figure S2

A Left lateral view of the present case

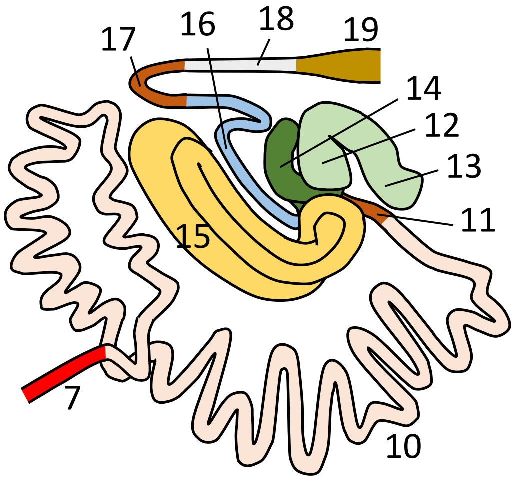

B Right lateral view of general cattle

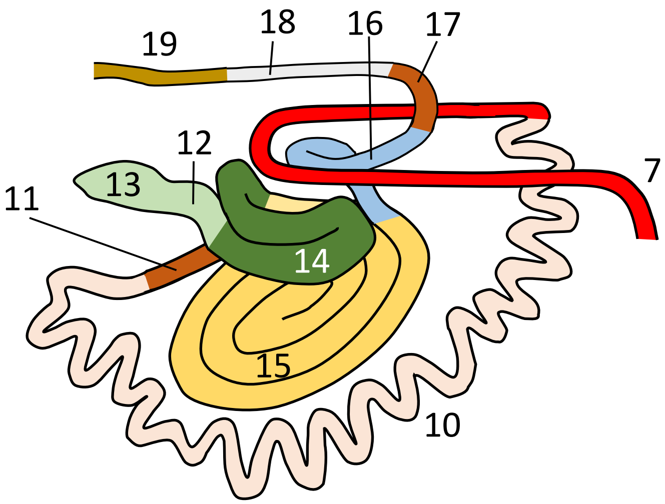

Figure S3

A Left lateral view of the present case

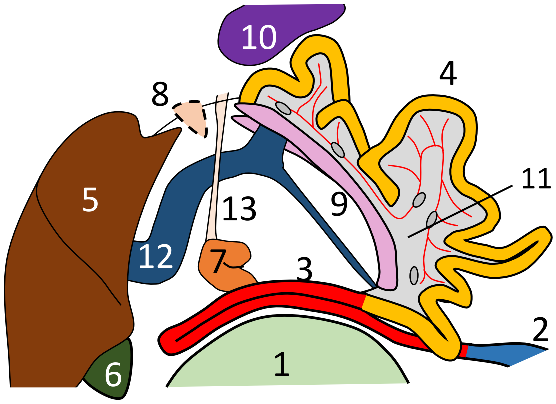

B Right lateral view of general cattle

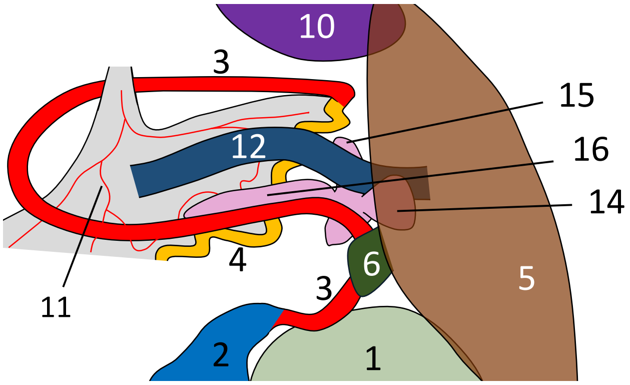

Figure S4
